# Artificial Intelligence and Neuroscience: Transformative Synergies in Brain Research and Clinical Applications

**DOI:** 10.3390/jcm14020550

**Published:** 2025-01-16

**Authors:** Razvan Onciul, Catalina-Ioana Tataru, Adrian Vasile Dumitru, Carla Crivoi, Matei Serban, Razvan-Adrian Covache-Busuioc, Mugurel Petrinel Radoi, Corneliu Toader

**Affiliations:** 1 Department of Neurosurgery, “Carol Davila” University of Medicine and Pharmacy, 020021 Bucharest, Romania; razvan.onciul@drd.umfcd.ro (R.O.); matei.serban2021@stud.umfcd.ro (M.S.); razvan-adrian.covache-busuioc0720@stud.umfcd.ro (R.-A.C.-B.); petrinel.radoi@umfcd.ro (M.P.R.); corneliu.toader@umfcd.ro (C.T.); 2 Neurosurgery Department, Emergency University Hospital, 050098 Bucharest, Romania; 3 Clinical Department of Ophthalmology, “Carol Davila” University of Medicine and Pharmacy, 020021 Bucharest, Romania; 4 Department of Ophthalmology, Clinical Hospital for Ophthalmological Emergencies, 010464 Bucharest, Romania; 5 Department of Morphopathology, “Carol Davila” University of Medicine and Pharmacy, 020021 Bucharest, Romania; 6 Emergency University Hospital, 050098 Bucharest, Romania; 7 Department of Computer Science, Faculty of Mathematics and Computer Science, University of Bucharest, 010014 Bucharest, Romania; crivoicarla02@gmail.com; 8 Department of Vascular Neurosurgery, National Institute of Neurovascular Disease, 077160 Bucharest, Romania; 9 Puls Med Association, 051885 Bucharest, Romania

**Keywords:** artificial intelligence, neuroscience, brain–computer interfaces, neuroimaging, neural signal processing, personalized medicine, neurological disorders, explainable AI, cognitive augmentation, multimodal data integration

## Abstract

The convergence of Artificial Intelligence (AI) and neuroscience is redefining our understanding of the brain, unlocking new possibilities in research, diagnosis, and therapy. This review explores how AI’s cutting-edge algorithms—ranging from deep learning to neuromorphic computing—are revolutionizing neuroscience by enabling the analysis of complex neural datasets, from neuroimaging and electrophysiology to genomic profiling. These advancements are transforming the early detection of neurological disorders, enhancing brain–computer interfaces, and driving personalized medicine, paving the way for more precise and adaptive treatments. Beyond applications, neuroscience itself has inspired AI innovations, with neural architectures and brain-like processes shaping advances in learning algorithms and explainable models. This bidirectional exchange has fueled breakthroughs such as dynamic connectivity mapping, real-time neural decoding, and closed-loop brain–computer systems that adaptively respond to neural states. However, challenges persist, including issues of data integration, ethical considerations, and the “black-box” nature of many AI systems, underscoring the need for transparent, equitable, and interdisciplinary approaches. By synthesizing the latest breakthroughs and identifying future opportunities, this review charts a path forward for the integration of AI and neuroscience. From harnessing multimodal data to enabling cognitive augmentation, the fusion of these fields is not just transforming brain science, it is reimagining human potential. This partnership promises a future where the mysteries of the brain are unlocked, offering unprecedented advancements in healthcare, technology, and beyond.

## 1. Introduction

### 1.1. Background and Motivation

Artificial Intelligence (AI) has emerged as a cornerstone in addressing the intricate challenges of neuroscience, a field focused on deciphering the complexities of the human brain. With approximately 86 billion neurons forming trillions of synaptic connections, the brain operates as a highly dynamic, non-linear system [1]. Understanding its functions—ranging from basic reflexes to higher-order cognition—relies on processing vast amounts of data, which span modalities such as neuroimaging, electrophysiology, and behavioral studies. Traditional analytical tools, while effective within limited scopes, often fall short when tasked with capturing the nuanced, multi-scale patterns embedded in neural data. This gap has driven the integration of AI as a critical tool for neuroscientific exploration [2].

AI technologies excel at uncovering patterns and relationships in complex, high-dimensional datasets. Among its methodologies, deep learning (DL) has proven particularly transformative, with neural network architectures such as convolutional neural networks (CNNs) enabling the precise analysis of neuroimaging data. These tools have been used to detect structural abnormalities linked to disorders like Alzheimer’s disease, offering diagnostic capabilities that surpass conventional techniques [3]. Similarly, recurrent neural networks (RNNs), designed to model temporal sequences, have enhanced our ability to interpret electrophysiological signals, making strides in areas like epilepsy monitoring, where predicting seizure onset can significantly improve patient outcomes [4].

Beyond its utility as an analytical tool, AI serves as a conceptual bridge, linking neuroscience’s understanding of biological intelligence to computational frameworks. Artificial neural networks (ANNs), inspired by the hierarchical organization of the brain, are modeled on principles such as Hebbian learning and synaptic plasticity. These networks, now foundational in AI, mimic the brain’s ability to process information efficiently through interconnected layers. Advances in neuromorphic computing, which seeks to replicate the spiking behavior of biological neurons in hardware, highlight the evolving feedback loop between neuroscience and AI [5]. This bidirectional relationship underscores how neuroscience not only benefits from AI but also inspires its evolution [6].

The practical implications of AI’s integration into neuroscience are vast. For instance, in brain–computer interfaces (BCIs), AI algorithms decode neural activity in real time, enabling paralyzed individuals to control external devices with their thoughts [7]. Such systems leverage cutting-edge neural decoders, translating signals from brain regions involved in motor control into precise commands [8]. In drug discovery, AI has streamlined the identification of therapeutic targets for neurological disorders by analyzing genetic, proteomic, and clinical data. Reinforcement learning (RL) algorithms have been particularly effective in optimizing molecular candidates, accelerating the development of treatments for conditions such as Parkinson’s disease (PD) [9].

However, the integration of AI into neuroscience is not without challenges. The interpretability of AI models remains a significant concern, particularly in fields like medicine, where understanding the rationale behind predictions is critical [10]. Neuroscience data, often characterized by noise and variability, further complicate model training and validation. Ethical considerations also loom large, especially in protecting patients’ data privacy and ensuring equity in the development and application of AI-driven tools [11].

Despite these challenges, the convergence of AI and neuroscience heralds a transformative era. By enabling more granular analyses of neural data, AI is uncovering insights that were previously inaccessible. Its application extends beyond research, driving innovations in clinical care and assistive technologies. This symbiosis between AI and neuroscience not only advances our understanding of the brain but also holds promise for improving the lives of millions affected by neurological conditions. As these fields continue to evolve together, their combined potential offers an unprecedented opportunity to reshape the future of neuroscience research and its real-world applications [12,13].

### 1.2. Scope and Objectives of the Review

The intersection of AI and neuroscience represents a transformative frontier, merging computational power with the complexity of brain science to address challenges once thought insurmountable. Neuroscience, driven by vast and diverse datasets from neuroimaging, electrophysiology, and genomics, has increasingly turned to AI to unlock new insights. AI, with its capacity to identify hidden patterns, model complex relationships, and make accurate predictions, offers unprecedented opportunities to deepen our understanding of the brain while advancing practical applications in research and medicine.

This review seeks to provide a detailed exploration of the convergence between AI and neuroscience, highlighting how these fields are not only advancing individually but also propelling each other forward. It begins by clarifying foundational concepts, bridging the gap for readers with diverse expertise. Core AI methodologies, including supervised, unsupervised, and reinforcement learning, are contextualized within neuroscience, demonstrating how these techniques address critical challenges, such as analyzing dynamic neural systems or decoding the subtle patterns embedded in noisy data.

A central focus of this review is the transformative impact of AI on neuroscience applications. From enhancing neuroimaging precision to advancing neural signal processing and predictive modeling, AI has enabled discoveries that were previously unattainable. For example, tools powered by AI now facilitate the accurate mapping of neural connectivity, the prediction of behavioral and cognitive outcomes, and the identification of early biomarkers for neurological disorders. These advancements are not only refining research techniques but also improving clinical interventions, enabling more personalized and effective care.

While the potential is vast, the integration of AI into neuroscience is not without its challenges. The variability and complexity of neuroscience data represent significant hurdles for AI algorithms, which require clean, high-quality, and standardized datasets to perform optimally. The “black-box” nature of many AI models poses additional concerns, particularly in medical applications where interpretability and trust are essential. Ethical considerations, including patient privacy, data security, and algorithmic bias, further complicate the landscape. This review examines these barriers, offering a critical perspective on how they might be addressed to ensure the responsible and effective use of AI.

Looking toward the future, this review explores emerging directions that promise to shape the next wave of progress at the AI–neuroscience interface. These include the development of explainable AI (XAI) models, which aim to improve transparency without sacrificing performance, and the integration of diverse data modalities—combining imaging, electrophysiological, and genetic data—to create a more holistic understanding of the brain. The application of AI in personalized neuroscience is also discussed, highlighting its potential to tailor treatments to individual neural profiles, paving the way for breakthroughs in precision medicine.

Ultimately, this review aims to inspire and inform, synthesizing recent advancements while identifying opportunities for innovation. By providing actionable insights and fostering interdisciplinary collaboration, it seeks to contribute to the ongoing evolution of both AI and neuroscience, ensuring that their combined potential is harnessed to address critical questions about the brain and its disorders. This is not merely a synthesis of past achievements but a call to action, encouraging researchers, clinicians, and technologists to continue pushing the boundaries of what AI and neuroscience can achieve together.

### 1.3. Structure of the Review

This review is organized to offer a coherent and in-depth exploration of the dynamic intersection between AI and neuroscience, guiding readers through foundational knowledge to cutting-edge applications and future possibilities. Each section is designed to build seamlessly upon the last, creating a flowing narrative that reflects the evolving relationship between these fields.

The journey begins with an Overview of AI, where the fundamental principles of AI are introduced. Key methodologies, such as machine learning (ML) and neural networks, are outlined in a way that highlights their relevance to neuroscience. By tracing the historical development of AI and emphasizing its current capabilities, this section provides a strong foundation for understanding its integration into neuroscience.

Following this, the Overview of Neuroscience delves into the essential concepts that underpin the study of the brain. It explores how neural structures and processes produce complex behaviors and cognitive functions while generating vast and diverse datasets. These include neuroimaging outputs, electrophysiological recordings, and genetic information, all of which present analytical challenges that AI is uniquely positioned to address. This section sets the stage for understanding the transformative potential of AI in neuroscience.

The next section, The Intersection of AI and Neuroscience, examines how these disciplines inspire and enhance one another. It discusses how biological principles from neuroscience have influenced AI architectures and, in turn, how AI has become an invaluable tool for decoding brain activity and modeling neural systems. By presenting this bidirectional relationship, this review illuminates the collaborative nature of progress at this interface.

At the heart of this review is the Applications of AI in Neuroscience, which explores real-world breakthroughs enabled by AI. This section highlights specific areas where AI has proven instrumental, from advancing neuroimaging analysis to improving neural signal processing and enabling brain–computer interfaces. It also examines how AI-driven computational modeling is shedding light on complex neural behaviors, supporting both research and clinical advancements.

To ground these discussions in practical outcomes, the Case Studies section presents significant examples of AI’s contributions to neuroscience. These include pioneering studies and collaborative projects that have reshaped the understanding and treatment of brain disorders. By analyzing these achievements, this review underscores the real-world impact of integrating AI into neuroscience.

The Challenges and Limitations section offers a critical perspective on the hurdles that remain. It explores the technical difficulties of managing diverse and noisy datasets, the interpretability challenges of AI models, and the ethical questions surrounding their application. This section highlights the importance of addressing these issues to ensure the responsible and equitable use of AI in neuroscience.

Our paper concludes with a forward-looking Future Directions and Opportunities section. Here, emerging trends such as XAI, multimodal data integration, and personalized neuroscience are explored. These advancements promise to push the boundaries of what AI and neuroscience can achieve together, paving the way for a new era of innovation.

By synthesizing these elements into a cohesive structure, this review provides a clear and engaging roadmap for understanding the evolving relationship between AI and neuroscience. Each section contributes to a deeper appreciation of the transformative potential at this intersection, while also identifying challenges that require collective effort to overcome.

## 2. Overview of AI

AI has emerged as a transformative force across scientific disciplines, with neuroscience standing out as one of its most promising applications. The unparalleled ability of AI to decipher complex, multidimensional data has revolutionized the way researchers analyze, interpret, and model brain functions [14]. By bridging the gap between raw data and actionable insights, AI offers a powerful toolkit for addressing the challenges inherent in neuroscience, a field characterized by its intricate structures and dynamic processes. This section provides a detailed yet fluid exploration of AI’s foundational principles, historical evolution, and specialized methodologies that have profoundly influenced neuroscience research [15].

### 2.1. Definition and Core Concepts

AI can be understood as a suite of computational systems designed to replicate human cognitive abilities such as reasoning, learning, and problem-solving. At its heart, AI relies on algorithms that adapt and improve with experience, rather than relying solely on pre-defined instructions. In neuroscience, AI serves as a crucial bridge, transforming massive, intricate datasets into meaningful patterns and predictions, ultimately advancing our understanding of the brain [16].

Two fundamental pillars of AI are ML and DL. ML focuses on teaching algorithms to identify patterns and relationships in data. One key approach is supervised learning, where models are trained on datasets labeled with known outcomes. In neuroscience, supervised learning is commonly used for diagnostic tasks, such as predicting Alzheimer’s disease (AD) progression from MRI data by identifying subtle structural changes [17]. On the other hand, unsupervised learning thrives in scenarios where the data lack clear labels, uncovering hidden structures within complex datasets. For example, clustering algorithms can reveal unique connectivity patterns across brain networks, shedding light on differences between healthy individuals and those with neurological conditions [18].

Deep learning, a specialized subset of ML, mimics the hierarchical nature of neural processing in the brain. ANNs, the backbone of deep learning, consist of interconnected layers that extract progressively complex features from input data. CNNs, for instance, are particularly effective in analyzing spatially structured data like neuroimaging scans, identifying lesions, or segmenting brain regions [19]. RNNs, another type of DL model, excel at capturing temporal dependencies, making them ideal for decoding time-series data such as EEG recordings. Transformers, a more recent innovation, leverage attention mechanisms to integrate diverse datasets, allowing for holistic analyses of brain function across multiple modalities [20].

### 2.2. Historical Development of AI

The story of AI is one of iterative innovation shaped by theoretical breakthroughs, technological advancements, and interdisciplinary applications. It began in the mid-20th century, with early AI systems focusing on symbolic reasoning and rule-based logic. These early models, such as the Logic Theorist and General Problem Solver, demonstrated the potential of computational reasoning but were constrained by their inability to handle real-world, unstructured data [21].

The 1980s marked a pivotal era with the introduction of artificial neural networks. Inspired by simplified representations of biological neurons, these networks promised to model more adaptive learning processes. However, their growth was initially stifled by limited computational resources and insufficient data availability. The advent of backpropagation, a method for fine-tuning the network’s parameters, rejuvenated interest in neural networks, setting the stage for the DL revolution [22].

In the late 2000s, a confluence of factors—including advances in computing power, the rise of big data, and refinements in training algorithms—ushered in a golden age for AI. CNNs became the standard for image-based tasks, transforming neuroimaging analyses by enabling precise segmentation and anomaly detection. RNNs expanded AI’s reach into sequential data, such as modeling neural activity over time [23]. Transformers, emerging in the past decade, have redefined AI’s capabilities, offering unparalleled flexibility in processing and synthesizing multimodal datasets. These advancements have firmly established AI as a cornerstone in neuroscience, enabling breakthroughs in understanding brain function and dysfunction [24].

These limitations were particularly evident in handling unstructured data, which lack a predefined organization and include formats such as text, images, and time-series data. For instance, textual data, like natural language, posed challenges due to their complex syntax, semantics, and contextual dependencies, making tasks like translation or sentiment analysis difficult for early systems [25]. Similarly, images, with their high-dimensional pixel arrays, required sophisticated methods to extract meaningful features such as edges or textures. Early AI models struggled to analyze such visual data effectively, hindering applications like image recognition or medical imaging [26]. Time-series data, such as EEG signals or financial trends, added another layer of complexity due to their sequential and dynamic nature. Capturing temporal dependencies within these datasets was beyond the capability of symbolic reasoning or rule-based systems [27].

The advent of backpropagation, a method for fine-tuning network parameters, rejuvenated interest in artificial neural networks, setting the stage for the deep learning revolution [28]. Backpropagation is considered revolutionary because it enables networks to learn by systematically adjusting weights through gradient descent, efficiently minimizing errors between predicted and actual outputs [29]. This breakthrough addressed the long-standing challenge of training multi-layered networks, unlocking their potential to model complex, non-linear relationships in data [30].

The development of machine learning, and later deep learning, transformed the landscape of unstructured data processing. CNNs addressed image processing challenges by extracting spatial features, while RNNs introduced mechanisms to retain memory, enabling the analysis of sequential data [31,32]. These innovations marked a pivotal step forward, bridging the gap between early models and the complexities of real-world data [33].

### 2.3. Types of AI Techniques Relevant to Neuroscience

AI’s success in neuroscience is rooted in its diverse methodologies, each tailored to address specific challenges posed by the complexity of the brain and its data. These approaches have empowered researchers to decode neural signals, predict cognitive outcomes, and simulate intricate brain networks [34].

Supervised learning is a cornerstone of AI applications in neuroscience. By training on labeled datasets, supervised models excel in diagnostic and predictive tasks. For instance, they are used to identify early biomarkers of neurodegenerative diseases, such as subtle structural changes in brain scans that signal cognitive decline. These models have also been deployed to classify neuronal activity patterns, helping researchers link brain function to specific behaviors or cognitive states [35].

Unsupervised learning, in contrast, is the method of choice for exploratory neuroscience. It is particularly valuable for uncovering latent patterns in neural connectivity or simplifying the complexity of large-scale datasets, such as single-cell gene expression profiles. By clustering similar data points or reducing dimensions, unsupervised algorithms have revealed novel insights into brain organization and function [36].

Unsupervised learning, in contrast, is the method of choice for exploratory neuroscience. It is particularly valuable for uncovering latent patterns in brain connectivity or simplifying the complexity of large-scale datasets, such as single-cell gene expression profiles [37]. Common algorithms include k-means clustering, which groups similar data points to reveal subtypes of neurons or patterns in brain connectivity, and principal component analysis (PCA), a dimensionality reduction technique often employed to identify dominant trends in high-dimensional neuroimaging or genomic data [38].

Another widely used method is t-distributed stochastic neighbor embedding (t-SNE), which is especially effective for visualizing high-dimensional data in two or three dimensions [39]. For example, t-SNE has been applied to single-cell transcriptomic data, enabling researchers to distinguish neuronal subtypes and map their spatial relationships. These algorithms play a crucial role in transforming complex, noisy datasets into interpretable formats, allowing neuroscientists to uncover hidden structures and generate new hypotheses [40].

RL offers a unique perspective, drawing inspiration from the brain’s reward systems. This technique trains AI agents to make sequential decisions by maximizing rewards, mirroring how organisms learn through trial and error [41]. Neuroscientists have used RL to model decision-making processes, decode neural signals linked to reward pathways, and understand how adaptive behaviors emerge from neural activity [42].

While RL has shown remarkable promise in neuroscience, its implementation is accompanied by unique technical challenges. One primary issue is the sample inefficiency of RL algorithms, which often require extensive interactions with an environment to learn optimal policies [43]. In neuroscience, this presents significant barriers, as data acquisition from participants or animal models is often time-intensive, ethically constrained, or limited in scale. Using high-fidelity simulations to generate synthetic data has emerged as a potential solution, but these models often fail to capture the full complexity of biological systems, limiting their applicability to real-world neural processes [44].

Another challenge is the computational intensity of RL, especially in applications requiring large-scale neural data, such as optimizing BCIs or simulating decision-making processes [45]. Training RL models on high-dimensional datasets like fMRI or EEG recordings can be resource-intensive, requiring advanced hardware such as GPUs or neuromorphic platforms to manage the memory and processing demands. Strategies such as parallelization or asynchronous learning can reduce the computational overhead, making RL more accessible to research teams with limited resources [46].

A notable obstacle is the generalization gap, where RL models struggle to adapt learned policies to new datasets or experimental conditions. Neural variability across individuals or sessions often leads to inconsistent model performance, such as a BCI trained for one individual’s neural patterns failing to generalize to another [47]. Promising approaches include meta-learning, which enables models to adapt rapidly to new conditions, and transfer learning, which allows pre-trained models to be fine-tuned for specific applications [48].

The interpretability of RL algorithms also poses a critical challenge, particularly in clinical contexts where understanding a model’s decisions is essential. This is especially relevant in areas like drug development, where RL is used to identify candidate molecules. Integrating XAI techniques, such as saliency maps or feature attribution, can make RL outputs more transparent and actionable for clinicians and researchers, fostering greater trust in these systems [49,50].

Finally, the reward design problem remains a significant limitation in neuroscience applications. RL models rely on carefully crafted reward signals to guide learning, but defining appropriate rewards can be complex in tasks like adaptive neural stimulations or optimizing therapeutic interventions [51]. Poorly designed reward structures can lead to unintended or suboptimal behavior. Techniques such as inverse reinforcement learning (IRL), which derives rewards from expert demonstrations, and multi-objective RL, which balances competing goals, are emerging as practical solutions to address this issue [52].

By addressing these challenges, RL can expand its utility in neuroscience, bridging the gap between theoretical potential and real-world applications. Continued innovation in simulation techniques, hardware efficiency, and model adaptability will be essential to overcome these barriers.

CNNs are indispensable in neuroimaging, where they enhance the analysis by automating tasks like tumor detection, segmentation of brain structures, and mapping functional connectivity [53]. These networks have also been adapted to analyze advanced imaging modalities, such as diffusion tensor imaging, to explore microstructural properties of the brain [54].

RNNs and their variants, such as long short-term memory (LSTM) networks, are vital for analyzing sequential neural data [55]. They excel in capturing dynamic relationships over time, enabling predictive modeling of neural activity, such as forecasting seizure events or understanding oscillatory brain activity during cognitive tasks [56].

Despite their successes, CNNs and RNNs face significant challenges in their application to neuroscience. One major limitation is their reliance on large, high-quality datasets to achieve optimal performance. In neuroscience, acquiring such datasets is challenging due to the labor-intensive nature of data collection and privacy constraints [57,58]. For instance, labeling EEG data requires extensive manual effort by experts, while neuroimaging datasets are often limited in availability, especially for rare neurological conditions.

Data standardization also represents a critical hurdle. Variability in EEG electrode configurations, MRI scanner settings, or participant demographics introduces inconsistencies that can hinder model generalization across studies. These discrepancies often necessitate substantial preprocessing and augmentation, adding complexity to the workflow [59].

High-dimensional data, such as MRI scans or long EEG recordings, further complicate computational requirements. Training these models on large-scale datasets demands significant memory and processing power, which can limit accessibility for smaller research teams. Additionally, overfitting is a persistent risk, particularly when models are trained on smaller datasets, reducing their reliability for unseen data [60].

Addressing these limitations requires innovative solutions, such as leveraging transfer learning to make use of pre-trained models and establishing standardized protocols for data collection and preprocessing. These advancements are vital to fully realizing the potential of CNNs and RNNs in neuroscience.

Transformers have emerged as a game changer in neuroscience, with their attention mechanisms allowing them to analyze complex relationships across entire datasets. This architecture has proven particularly effective in integrating multimodal data, combining neuroimaging data, genetic profiles, and behavioral metrics to provide a more comprehensive understanding of brain function and disease mechanisms [61].

Generative models, including Variational Autoencoders (VAEs) and Generative Adversarial Networks (GANs), offer creative solutions for generating realistic neural activity or synthetic datasets [62]. These techniques are invaluable in scenarios where real-world data are scarce, such as for rare neurological conditions, and provide a sandbox for testing hypotheses or training other AI systems [63].

While generative models such as GANs and VAEs have revolutionized the creation of synthetic datasets, their use is accompanied by challenges that must be addressed for broader applicability in neuroscience. A key concern is the accuracy and biological validity of synthetic data [62]. Although these models aim to replicate the statistical properties of real data, their output can lack subtle patterns inherent in complex neural datasets. For instance, synthetic fMRI data generated from limited training datasets may fail to reflect the variability and noise present in real-world data, potentially introducing biases into downstream analyses [64].

Another significant issue is the sim-to-real gap, where models trained on synthetic data struggle to perform well on real-world tasks due to discrepancies in data distributions. This gap can limit the generalizability of models trained solely on synthetic datasets [65]. A promising solution is the development of hybrid datasets, which combine synthetic data with smaller amounts of real data to enhance training quality while mitigating the reliance on large, hard-to-obtain datasets. Domain adaptation techniques are also being explored to align synthetic data more closely with real-world distributions, improving model robustness [66].

Furthermore, synthetic data use raises concerns about validation and clinical trust. In applications such as diagnostic tool development, models trained on synthetic data may face scrutiny regarding their reliability and safety [67]. Establishing rigorous validation pipelines, including independent benchmarking and testing against diverse real-world datasets, is essential to ensure clinical acceptance. Collaborative efforts between AI researchers and neuroscientists can help refine synthetic data pipelines and align them with the standards required for clinical and translational applications [68].

Despite these challenges, synthetic data remain a transformative tool for neuroscience, particularly in scenarios where real-world data are scarce or ethically constrained. Addressing these issues will unlock the full potential of generative models, allowing them to play a pivotal role in accelerating discoveries and advancing AI-driven neuroscience research [69].

These methodologies have transformed neuroscience, enabling researchers to navigate the complexity of brain data with precision and efficiency. By leveraging AI’s versatility, neuroscientists are not only unraveling the mysteries of the brain but also developing tools and insights that promise to revolutionize research and clinical care [70].

## 3. Overview of Neuroscience

Neuroscience is the intricate study of the nervous system, striving to uncover the principles that govern brain function, cognition, behavior, and disease. With its vast scope, the field draws on diverse disciplines, including molecular biology, neuroimaging, and behavioral science, weaving these perspectives into a cohesive effort to understand one of the most complex systems known to science. Recent technological breakthroughs and computational advances, particularly the integration of AI, are accelerating discoveries, allowing researchers to tackle long-standing mysteries with newfound clarity [14,71,72].

### 3.1. Fundamental Concepts in Neuroscience

The nervous system is a marvel of complexity, comprising billions of neurons and glial cells interconnected in dynamic and adaptive networks. Neurons, the brain’s primary signaling units, communicate through electrical impulses that travel along axons and trigger the release of neurotransmitters at synapses. These chemical signals bridge the gap between neurons, facilitating the flow of information. Meanwhile, glial cells, traditionally viewed as support elements, have emerged as active players, regulating synaptic activity, maintaining neural homeostasis, and orchestrating responses to injury [73].

The brain’s adaptability, known as neuroplasticity, is one of its defining features. This ability to reorganize and reshape itself in response to learning, environmental changes, or damage occurs at multiple levels [74]. Synaptic plasticity, driven by mechanisms like long-term potentiation (LTP) and long-term depression (LTD), strengthens or weakens connections based on activity, forming the foundation of learning and memory.

Among the mechanisms underlying neuroplasticity, LTP and LTD are pivotal processes that enable the strengthening or weakening of synaptic connections. These mechanisms are studied through precise experimental techniques designed to capture synaptic changes at molecular, cellular, and network levels [75]. Electrophysiological methods, such as patch-clamp and field recordings, are commonly employed to monitor synaptic responses during LTP and LTD induction. These approaches, often applied to brain slices or cultured neurons, allow researchers to measure synaptic strength in real-time and to assess the effects of specific stimulation protocols, such as high-frequency stimulation (HFS) for LTP or low-frequency stimulation (LFS) for LTD [76].

To explore these mechanisms in living systems, in vivo techniques such as calcium imaging and two-photon microscopy are increasingly utilized. These methods enable the visualization of synaptic activity and structural remodeling, such as dendritic spine changes, within intact neural circuits. By capturing these processes during behavior or environmental interactions, researchers can connect LTP and LTD to their functional roles in memory and learning [77,78].

However, studying LTP and LTD presents several challenges. Electrophysiological recordings, while precise, are inherently invasive and often restricted to small neuronal populations, limiting insights into broader network interactions [79]. In vivo imaging, though non-invasive, faces difficulties with signal-to-noise ratios and spatial resolution, particularly in deeper brain structures [80]. Additionally, variability in experimental protocols, including differences in animal models, synapse types, and stimulation parameters, often complicates reproducibility and cross-study comparisons [81].

Advancing the study of LTP and LTD requires the standardization of protocols and the integration of complementary approaches. Computational modeling offers a promising avenue for bridging scales, enabling researchers to simulate plasticity mechanisms and interpret experimental data more effectively. By addressing these challenges, researchers can further unravel the roles of LTP and LTD in shaping adaptive neural circuits and behavior [82,83,84].

Beyond the synapse, structural plasticity involves the remodeling of dendrites and axons, as seen during recovery from brain injury or the acquisition of new skills [85]. Recent advances in imaging have unveiled nanoscale transformations within synaptic structures, shedding light on how experiences shape the brain.

While specific brain regions are specialized for particular tasks—such as the hippocampus for memory encoding or the visual cortex for processing visual stimuli—these regions seldom act in isolation. Instead, they collaborate as part of dynamic networks, coordinating activity to perform complex functions [86]. Functional MRI (fMRI) studies have revealed key networks like the default mode network (DMN), which is active during introspection and memory retrieval, and the salience network, which guides attention to critical external stimuli. While fMRI has significantly advanced our understanding of functional brain networks, it has inherent limitations that must be considered. One key challenge is its limited temporal resolution, as fMRI relies on measuring blood-oxygen-level-dependent (BOLD) signals, which reflect metabolic changes rather than the millisecond-scale electrical activity of neurons [87]. This delay makes it difficult to capture rapid, dynamic interactions between networks, particularly during complex cognitive tasks [88].

Additionally, fMRI’s spatial resolution, although effective for identifying large-scale networks, is less precise when examining finer details, such as the microcircuitry within specific brain regions. Artifacts near air–tissue interfaces, such as those around the orbitofrontal cortex, can further reduce accuracy [89,90].

Another limitation lies in the correlational nature of functional connectivity analyses. While these methods reveal statistical associations between regions, they cannot establish causal relationships. Effective connectivity techniques, like dynamic causal modeling (DCM), address this gap but often require complex computational modeling and assumptions, which can vary across studies [91,92].

To overcome these challenges, researchers increasingly integrate fMRI with complementary methods like EEG and MEG. These multimodal approaches combine fMRI’s strength in spatial resolution with the high temporal precision of electrophysiological techniques, offering a more holistic view of network dynamics [93,94].

Interactions between brain networks, such as the DMN and the salience network, are crucial for coordinating complex cognitive processes. These interactions are often studied using functional connectivity analyses derived from fMRI [95]. Functional connectivity examines statistical correlations between neural activities in different regions, revealing how networks collaborate during rest or task engagement. For instance, during attention-demanding tasks, the salience network identifies relevant external stimuli and facilitates the transition from the internally focused DMN to task-oriented networks like the frontoparietal network [96].

To uncover directional influences between networks, researchers employ effective connectivity techniques, such as DCM and Granger causality. These methods help them map how activity in one network influences another, showing, for example, how the salience network modulates DMN activity to optimize cognitive performance [97].

Complementary insights are provided by EEG and MEG, which capture network interactions at higher temporal resolutions. Cross-frequency coupling (CFC), where slower oscillations influence faster rhythms, has been observed between the DMN and salience network during tasks requiring attention shifts. This synchronization supports the rapid reconfiguration of brain states essential for cognitive flexibility [98].

Studying network interactions comes with challenges, such as the computational complexity of connectivity models and the need for rigorous statistical controls to mitigate spurious findings [99]. Moreover, differences in imaging modalities and analytical assumptions can yield inconsistent results across studies. Multimodal approaches that combine fMRI, EEG, and MEG are increasingly used to overcome these limitations, offering a more comprehensive view of how brain networks dynamically interact to support cognition. Understanding these networks has shifted the focus from isolated regions to the intricate choreography of brain-wide interactions [100].

Oscillatory activity, or brain rhythms, is another cornerstone of neuroscience. These rhythmic patterns of neural firing, categorized by frequency bands such as theta, alpha, and gamma, synchronize activity across regions, enabling processes like attention, sensory integration, and working memory [101]. Cross-frequency coupling, where slower oscillations regulate faster ones, has been identified as a mechanism for coordinating information flow during cognitive tasks. Disruptions in these rhythms are increasingly linked to neurological and psychiatric conditions, offering new avenues for therapeutic interventions [102].

### 3.2. Current Challenges in Neuroscience Research

Despite remarkable progress, neuroscience remains a field defined by its challenges. One of the most significant hurdles is bridging the gap between molecular-level discoveries and systems-level understanding. While techniques like single-cell RNA sequencing (scRNA-seq) and optogenetics provide detailed insights into cellular functions, linking these findings to whole-brain dynamics and behavior is a complex and ongoing endeavor. This challenge is compounded by the multiscale nature of neural activity, which spans nanometer-sized synaptic changes to interactions between entire brain regions [103,104].

Temporal resolution also poses a persistent challenge. fMRI offers exquisite spatial detail but captures neural activity indirectly and with delays due to the hemodynamic response. Conversely, techniques like EEG and MEG provide millisecond precision but lack detailed spatial localization. Hybrid approaches, such as simultaneous EEG-fMRI recordings, are emerging as promising solutions, though they require advanced computational frameworks for effective integration [105].

Another layer of complexity arises from the inherent variability of the nervous system. Individual differences in brain anatomy, connectivity, and activity patterns are shaped by genetics, development, and life experiences. For example, even in individuals with the same neurological condition, the underlying neural mechanisms may differ significantly, complicating the development of generalized models [106]. Personalized neuroscience, which combines data from neuroimaging, genomics, and behavior, offers a way forward, tailoring models to account for these variations [107].

The translation of neuroscience findings into clinical practice remains an ongoing challenge. Despite major strides in understanding diseases like Alzheimer’s disease and epilepsy, effective treatments remain elusive. A critical bottleneck is the lack of reliable biomarkers that can predict disease onset, progression, or the response to therapies. Ethical concerns surrounding emerging technologies, such as BCIs and neuromodulation techniques, also present challenges. These innovations raise important questions about privacy, autonomy, and equitable access, emphasizing the need for careful ethical considerations as the field advances [108].

### 3.3. Data Types and Sources in Neuroscience

The diversity of data sources in neuroscience reflects the complexity of the brain and the multifaceted approaches needed to study it. Each modality provides unique insights, and their integration is increasingly essential for advancing our understanding of the nervous system [109].

Neuroimaging has revolutionized how researchers study the brain. Structural MRI offers detailed maps of anatomy, revealing changes associated with aging, trauma, or disease. fMRI tracks activity-related changes in blood flow, providing insights into how brain regions communicate during tasks or at rest. Diffusion tensor imaging (DTI) traces white matter pathways, illuminating the structural connections that support neural communication. Positron emission tomography (PET), often paired with MRI, enables researchers to study molecular processes, such as the buildup of amyloid plaques in AD or dopamine dysregulation in Parkinson’s disease [110,111,112].

Electrophysiological techniques provide an unparalleled view of neural activity at high temporal resolution. Non-invasive methods like EEG and MEG capture electrical and magnetic signals generated by neuronal ensembles, making them invaluable for studying oscillatory activity or event-related responses [113]. Invasive approaches, such as multi-electrode arrays and patch-clamp recordings, allow researchers to measure the activity of individual neurons or small networks with exceptional precision, offering a window into neural coding during complex behaviors [114].

Genomic and molecular approaches are transforming neuroscience at the cellular level. scRNA-seq has revealed the diversity of neuronal and glial cell types, linking gene expression patterns to specific roles within circuits. Genome-wide association studies (GWASs) have identified genetic variants associated with neuropsychiatric disorders, providing targets for therapeutic development. These molecular insights are increasingly integrated with functional data, offering new perspectives on how genetic factors shape neural systems [115,116].

Behavioral data remain vital for understanding how the brain drives real-world actions. Advances in wearable technologies and virtual reality environments have enabled researchers to study behaviors like navigation, decision-making, and social interaction in more naturalistic settings. These approaches provide richer datasets, allowing researchers to link neural activity to ecologically relevant behaviors [117,118].

Emerging technologies are redefining how data are collected and interpreted in neuroscience. Volumetric calcium imaging enables the real-time tracking of neural activity across large populations of neurons, while whole-brain clearing methods, such as CLARITY, provide detailed three-dimensional maps of neural circuits [119,120]. Hybrid modalities, like optogenetics–functional MRI, allow researchers to establish causal links between neural activation patterns and behaviors, bridging the gap between observation and intervention [121].

The integration of these diverse datasets presents challenges but also unparalleled opportunities. AI-powered approaches are increasingly critical for managing and analyzing multimodal data, identifying subtle patterns, and creating predictive models that span scales from molecules to behavior [2]. For example, ML algorithms are being used to combine PET imaging, EEG signals, and genetic data to predict the progression of neurological diseases, offering new avenues for personalized medicine and early intervention [122].

## 4. The Intersection of AI and Neuroscience

The intersection of AI and neuroscience represents a vibrant and transformative collaboration, one that is reshaping our understanding of both fields. Neuroscience, with its profound insights into the workings of the brain, provides inspiration and biological grounding for computational models, while AI offers the analytical power to tackle the complexities of neural data and simulate brain processes [13]. This relationship has unlocked new possibilities for exploring cognition, uncovering the roots of neurological disorders, and even designing smarter and more adaptive technologies. Here, we explore how these disciplines connect, influence one another, and give rise to innovations that are changing the landscape of science and technology [16].

### 4.1. Theoretical Connections

AI, as we know it today, owes much of its existence to the pioneering work of neuroscientists who sought to decode the inner workings of the brain. The first ANNs were inspired by the structure of biological neurons, simplifying their properties into mathematical models. Early efforts, such as the McCulloch–Pitts neuron, introduced the concept of threshold-based activation, where a neuron “fires” when inputs cross a certain threshold. Though rudimentary by modern standards, these ideas laid the groundwork for decades of innovation [123].

Hebbian learning, often summarized as “neurons that fire together wire together”, added a new dimension to AI. This principle of strengthening connections through repeated co-activation became a cornerstone of learning algorithms, influencing everything from unsupervised learning models to contemporary neural networks [124,125]. A more nuanced understanding emerged with spike-timing-dependent plasticity (STDP), which emphasized the importance of timing in shaping synaptic strength. This biologically inspired concept paved the way for spiking neural networks (SNNs), a class of models that incorporate the temporal dynamics of neural activity for greater computational realism [126].

Perhaps no discovery in neuroscience has influenced AI more profoundly than the brain’s hierarchical processing of sensory information [127]. Hubel and Wiesel’s Nobel-winning studies of the visual cortex revealed how neurons respond to progressively complex features, from edges to patterns. This inspired the architecture of CNNs, which excel at tasks like image recognition and neuroimaging analysis [128]. The brain’s influence on AI extends even further with RL, where studies of dopamine pathways and reward prediction errors have informed algorithms that replicate the brain’s ability to learn through feedback and adapt to changing environments [129].

**Figure 1:**
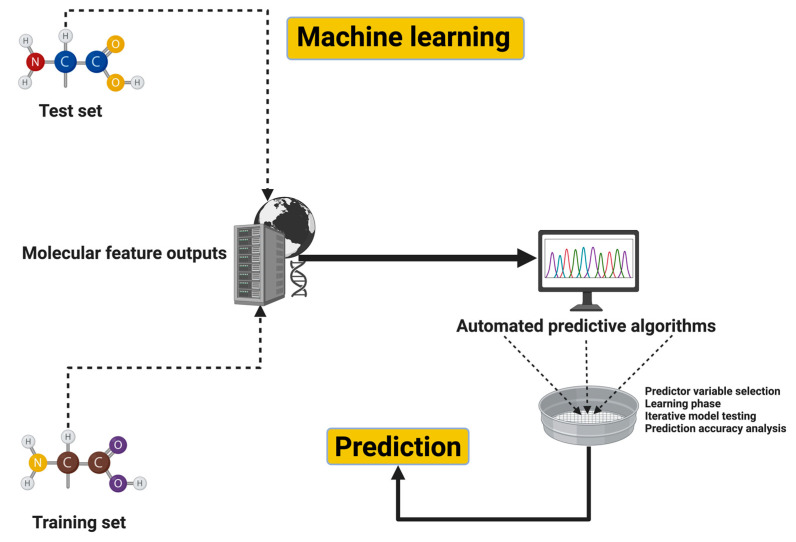
Workflow demonstrating how molecular feature outputs are processed through machine learning algorithms, culminating in predictions based on trained models.

Figure 1 illustrates the workflow for integrating molecular feature outputs into a machine learning pipeline for predictive analysis. The process begins with two datasets: a training set, which is used to build the model, and a test set, which is reserved for evaluating its performance. Molecular features or descriptors are extracted from these datasets using computational tools, generating structured input variables for machine learning algorithms. These features are then processed through automated predictive algorithms, involving critical steps such as attribute selection, model training, cross-validation, and iterative model testing. These steps ensure that the model effectively learns patterns and relationships within the data. Once trained, the model is applied to the test set to generate predictions, which may represent properties or behaviors of the molecules. The results, often visualized as graphs or numerical outputs, provide valuable insights for decision-making and applications in fields such as drug discovery and materials science. This workflow exemplifies how machine learning integrates data-driven approaches with molecular analyses to deliver accurate and actionable predictions.

### 4.2. AI as a Tool for Neuroscience

AI has transformed neuroscience by providing tools capable of unraveling the immense complexity of the brain. Modern neuroscience generates vast and intricate datasets—from high-resolution brain scans to recordings of thousands of neurons firing simultaneously. AI brings the ability to analyze, interpret, and model these data in ways that were previously unimaginable [130].

In neuroimaging, AI has revolutionized image analysis. DL models, particularly CNNs, have automated tasks like segmenting brain structures, detecting subtle abnormalities, and identifying disease biomarkers. For example, AI has been used to detect cortical thinning in MRI scans, an early marker of Alzheimer’s disease, with a precision that matches or exceeds expert radiologists [131]. Functional imaging has also benefited from AI, with ML algorithms decoding patterns of brain activity associated with specific cognitive states or diseases, such as schizophrenia and autism [132].

Electrophysiology, which captures the electrical activity of the brain, has seen similar advancements. Temporal data, such as EEG recordings or neural spiking activity, pose challenges due to their high dimensionality and complexity [133]. RNNs and transformers have become indispensable for analyzing these datasets, enabling breakthroughs like predicting seizures in epilepsy patients or decoding motor intentions for BCIs.

High-resolution EEG data offer valuable insights into neural activity, particularly in conditions like epilepsy, but their analysis is often complicated by noise and artifacts. Non-neural signals, such as muscle activity, eye blinks, and external interference, can obscure critical patterns, requiring robust preprocessing to improve the data quality before AI models are applied [134].

Preprocessing steps are critical to ensure reliable results. Techniques like bandpass filtering isolate neural signals within key frequency ranges while removing unwanted high-frequency noise and electrical artifacts. Independent component analysis (ICA) is commonly used to separate neural activity from non-neural artifacts, such as eye blinks or muscle contractions [135]. Wavelet-based denoising further refines the data by selectively suppressing noise while preserving essential features of neural activity. Additionally, normalization is employed to minimize variability across channels and sessions, ensuring that AI models can generalize effectively across datasets [136].

AI models further enhance noise handling by leveraging advanced architectures. Attention mechanisms in transformers and RNNs allow these models to focus on meaningful temporal or spatial features, effectively filtering out less relevant segments [137]. Convolutional layers in deep learning models extract robust spatial patterns that remain consistent despite residual noise, while data augmentation—such as adding synthetic noise during training—enhances model robustness by teaching algorithms to differentiate between true neural activity and artifacts [138].

These models are also adaptable to different seizure types and patient-specific patterns. Personalized AI models trained on individual EEG data can capture unique characteristics of a patient’s seizure activity, improving accuracy [139]. For broader applications, multi-label classification approaches enable models to differentiate between seizure types, such as focal and generalized seizures, based on distinct neural signatures. Transfer learning allows pre-trained models to be fine-tuned for specific patients with minimal additional data, ensuring both scalability and precision [140].

AI systems incorporating these methods have demonstrated exceptional accuracy in seizure detection, with sensitivity rates exceeding 90% in clinical studies. This precision reduces false-positive rates and facilitates real-time applications, such as closed-loop therapeutic systems where seizure detection triggers immediate interventions, like neural stimulation [141]. By addressing both noise and adaptability, AI offers a transformative approach to epilepsy management, combining diagnostic precision with personalized care.

AI-driven spike sorting has further streamlined the analysis of neural recordings, allowing researchers to isolate signals from individual neurons in complex datasets [142].

AI is not just a tool for analyzing data, it is also a powerful ally in modeling the brain itself. Graph neural networks (GNNs) have been used to simulate large-scale brain networks, revealing how the brain’s structure influences its functional dynamics [143]. Computational models informed by RL have provided insights into decision-making processes, mirroring the neural dynamics underlying behaviors like reward-seeking and risk assessment. These models help neuroscientists test hypotheses and refine theories of brain function, offering a deeper understanding of how neural circuits work together to produce cognition and behavior [144].

AI’s integration into clinical neuroscience has yielded transformative advancements in diagnostics and therapy, with several real-world implementations demonstrating its impact.

In neuroimaging, AI has significantly enhanced the detection of early disease markers. One notable application involves the use of CNNs to analyze MRI scans for cortical thinning, an early indicator of Alzheimer’s disease [64]. A landmark study demonstrated that an AI system achieved a diagnostic sensitivity of 94% and specificity of 92%, outperforming many radiologists in accuracy and processing speed [145].

In patients with AD, the DMN is among the earliest functional networks to show impairment, with disruptions closely linked to amyloid-beta (Aβ) plaque accumulation and tau protein tangles. The DMN, which includes the posterior cingulate cortex, precuneus, and medial prefrontal cortex, is essential for memory and self-referential thinking. Its high baseline activity and energy demands make it particularly vulnerable to Aβ toxicity, which interferes with synaptic signaling and disrupts network connectivity.

Research has shown that in the early stages of AD, Aβ accumulation in DMN regions correlates with hyperconnectivity, as the brain attempts to compensate for emerging damage. Over time, as tau pathology spreads along DMN connections—beginning in the entorhinal cortex and extending to the hippocampus—this hyperconnectivity transitions to hypoconnectivity, reflecting synaptic failure and neuronal loss [146]. Advanced imaging studies have demonstrated that tau deposition in DMN nodes is associated with reduced glucose metabolism and impaired functional connectivity, directly linking the molecular pathology to cognitive decline [147].

AI has been pivotal in studying these complex interactions. Machine learning models have been applied to combined PET and fMRI datasets, enabling the identification of subtle connectivity changes associated with amyloid and tau pathology [148]. For instance, AI algorithms have successfully predicted the progression of AD by quantifying how molecular disruptions influence DMN connectivity. Unsupervised clustering techniques have also been employed to stratify patients into subgroups based on molecular and functional profiles, supporting personalized predictions of the disease trajectory [149].

Furthermore, AI simulations have been used to assess the potential impacts of therapeutic interventions targeting amyloid or tau. These models provide valuable insights into how treatments might preserve DMN connectivity and mitigate cognitive decline, emphasizing AI’s role not only in diagnostics but also in guiding intervention strategies [150].

By automating the identification of subtle structural changes that are often missed during manual review, AI enables clinicians to diagnose Alzheimer’s disease earlier, offering opportunities for timely therapeutic interventions and improved patient outcomes [151].

Electrophysiology has also benefited from AI-driven advancements, particularly in BCIs. One clinical trial involved patients with tetraplegia using a BCI powered by RNNs to control robotic prosthetic limbs. The AI system translated neural activity patterns from the motor cortex into precise motor commands, allowing participants to perform tasks such as grasping objects and self-feeding [152]. Over the course of the trial, patients demonstrated significant improvements in task completion rates and accuracy, highlighting the potential of AI-enhanced BCIs to restore functional independence in individuals with severe motor impairments [152].

In mental health, AI-driven digital therapeutics are redefining how psychological support is delivered. Platforms such as Woebot and Wysa employ natural language processing (NLP) algorithms to provide cognitive–behavioral therapy (CBT) interventions via chat-based interfaces [153,154]. A randomized controlled trial involving over 1000 participants found that users of these platforms experienced a 25% reduction in anxiety symptoms and a 30% improvement in mood scores over eight weeks, outcomes comparable to traditional face-to-face therapy for patients with mild to moderate cases [155]. These systems address the growing demand for mental health services, offering scalable and accessible solutions for underserved populations.

AI has also accelerated drug discovery in neuroscience, particularly for neurodegenerative disorders. By analyzing vast molecular datasets and predicting drug–target interactions, AI systems can identify promising therapeutic candidates with unprecedented speed. In one case, an ML platform screened billions of compounds and identified a candidate drug for Alzheimer’s disease in just six months—a process that would typically take several years using conventional methods. This drug is now undergoing preclinical trials, demonstrating AI’s ability to expedite the development pipeline and address critical therapeutic gaps in neurology [156].

These case studies exemplify how AI is bridging the gap between research and clinical practice, improving patient outcomes across diverse medical domains. However, challenges remain, including ensuring the generalizability of AI models across populations, integrating these tools into existing clinical workflows, and navigating regulatory approval processes. Addressing these issues will be key to unlocking AI’s full potential in clinical neuroscience [157].

Molecular neuroscience has also embraced AI, particularly in the analysis of scRNA-seq data. ML algorithms have identified previously unrecognized neuronal subtypes and illuminated their roles within complex circuits. By linking molecular diversity to functional outcomes, these tools are reshaping our understanding of how genetic factors influence brain development and dysfunction [158,159].

Despite the transformative potential of ML in analyzing single-cell data, several challenges constrain its utility. A primary issue is the high dimensionality of scRNA-seq datasets, which often include thousands of gene expression measurements per cell [160]. This complexity can lead to overfitting, where models capture noise instead of meaningful patterns, particularly in smaller datasets or when rare cell subtypes are underrepresented. While dimensionality reduction methods, such as PCA and uniform manifold approximation and projection (UMAP), are widely used, they can oversimplify the data, potentially masking subtle biological variations [161].

Another challenge lies in the variability between datasets. Differences in experimental protocols, sequencing platforms, and sample preparation can introduce batch effects that obscure true biological signals. For instance, cells with similar expression profiles may cluster separately if derived from different experiments, complicating cross-study comparisons [162]. Methods like Harmony and ComBat have been developed to correct for these effects, but they require significant computational resources and careful optimization to avoid unintended distortions of the data [163].

The interpretability of ML models is also a major limitation. Advanced models like neural networks and ensemble methods often function as black boxes, making it difficult to discern the biological features driving predictions. This lack of transparency hinders the identification of novel pathways or cell subtypes. Efforts to incorporate XAI tools, such as integrated gradients and SHAP (Shapley Additive Explanations), are underway but have yet to see widespread application in single-cell research [164].

Furthermore, the scarcity of labeled datasets poses a significant barrier to training robust supervised ML models. Annotating single-cell data is labor-intensive and typically requires expert validation, which is particularly challenging for rare or poorly characterized cell populations. Consequently, many analyses rely on unsupervised or semi-supervised approaches, which may struggle to resolve complex or overlapping cell states without sufficient prior knowledge [165].

Finally, the computational demands of single-cell data analysis present practical challenges. Clustering, trajectory inference, and cell–cell interaction predictions often involve resource-intensive algorithms, making large-scale analyses inaccessible to research teams without a high-performance computing infrastructure. Efforts to optimize these workflows for scalability and efficiency remain an active area of research.

Addressing these challenges will require innovative solutions, such as standardized preprocessing pipelines, improved XAI frameworks, and collaborative initiatives for data sharing and model validation. By overcoming these limitations, ML has the potential to further unlock the complexities of single-cell data and enhance our understanding of molecular neuroscience.

### 4.3. Neuroscience-Inspired AI Innovations

While AI has empowered neuroscience, the influence flows both ways. Many of AI’s most significant breakthroughs have drawn inspiration directly from the brain. This interplay has led to the development of systems that are not only more powerful but also more efficient, adaptive, and interpretable [166].

SNNs are a prime example of how neuroscience inspires AI. Unlike traditional ANNs, which process information as continuous activations, SNNs replicate the brain’s discrete spiking behavior. This temporal fidelity makes them highly efficient, particularly when deployed on neuromorphic hardware that mimics the architecture of biological neurons [167]. SNNs are being explored for applications ranging from real-time robotics to energy-efficient computing, areas where the brain’s resourcefulness is an invaluable model [168].

The brain’s ability to integrate information across multiple senses has inspired multimodal AI systems. These models combine data streams, such as visual, auditory, and textual inputs, to generate richer and more contextually aware outputs. In autonomous vehicles, for instance, multimodal AI mimics the brain’s ability to integrate sensory information, enabling safer and more reliable navigation [169].

RL continues to evolve under the influence of neuroscience. Insights into how animals balance exploration and exploitation have informed algorithms that train AI agents to optimize behavior in dynamic environments. For example, deep RL models have been used to develop agents capable of solving complex problems, such as playing advanced strategy games or managing logistics in supply chains [170,171].

Another area of growth is continual learning, inspired by the brain’s capacity to retain and integrate knowledge across experiences. Unlike traditional AI models, which often suffer from catastrophic forgetting, neuroscience-informed algorithms incorporate mechanisms like memory consolidation and synaptic plasticity to enable incremental learning [172]. These systems are critical for applications requiring adaptability, such as personalized healthcare or autonomous systems that operate in changing environments [173].

Finally, XAI, a field aimed at making ML models transparent and interpretable, has drawn from cognitive neuroscience. By mimicking the brain’s mechanisms for attention and decision-making, these models prioritize relevant features and provide rationales for their outputs [174]. This interpretability is essential in applications like medical diagnostics, where trust and accountability are paramount [175].

The ongoing dialogue between AI and neuroscience is driving innovations that benefit both disciplines. Neuroscience inspires more flexible, efficient, and biologically plausible AI models, while AI accelerates discoveries in brain science, offering tools to analyze data, test hypotheses, and model complex processes. Together, these fields are pushing the boundaries of what is possible, laying the groundwork for new breakthroughs in understanding intelligence—both natural and artificial [14].

## 5. Applications of AI in Neuroscience

AI has revolutionized neuroscience, catalyzing groundbreaking advancements in research, diagnosis, and treatment. By enabling more precise data analysis, uncovering novel insights, and offering innovative solutions, AI is redefining how we understand and address the complexities of the brain [176,177]. This section integrates the novel and highly relevant contributions of AI to neuroimaging, neural signal processing, brain–computer interfaces, computational modeling, drug discovery, and cognitive–behavioral studies. These applications are enriched with perspectives from high-impact studies to showcase the transformative potential of AI in neuroscience.

### 5.1. Neuroimaging Analysis: Beyond Conventional Boundaries

AI has redefined neuroimaging by enabling the deep integration of multimodal data, automated segmentation, and early detection of subtle biomarkers. The convergence of structural and functional data through AI is shedding light on disease mechanisms and improving diagnostics.

Advancing Multimodal Imaging Integration.

AI’s ability to merge diverse imaging modalities is addressing a long-standing challenge in neuroscience: linking structural, functional, and molecular brain data. A highly cited application demonstrated how DL models integrated structural MRI with fMRI and PET to predict cognitive decline in preclinical AD models [178]. By correlating amyloid deposition PET with fMRI, these models offered unparalleled predictive accuracy, enabling earlier interventions [1].

Dynamic Functional Connectivity Analysis.

fMRI has benefited significantly from AI-powered GNNs, which map dynamic brain connectivity patterns over time. These networks have revealed activity disruptions in patients with conditions like schizophrenia, where altered connectivity in the default mode and salience networks impairs executive function [179]. Novel temporal analysis techniques, such as RNNs, have captured how functional interactions evolve during memory encoding and retrieval tasks, offering insights into conditions like mild cognitive impairment [180]. Table 1 summarizes key studies demonstrating AI’s pivotal role in advancing neuroimaging technologies and methodologies.

### 5.2. Neural Signal Processing: Unlocking Temporal Complexity

AI’s impact on neural signal processing has been transformative, particularly in its ability to decode intricate temporal dynamics and uncover new insights into brain function. Cutting-edge approaches like transformers are redefining how we analyze continuous neural signals.

Decoding Oscillatory Dynamics with Transformers.

Transformers, originally developed for natural language processing, are revolutionizing the analysis of EEG and MEG data [190]. These models excel at capturing long-range dependencies and interactions in neural oscillations, such as theta–gamma coupling, which plays a crucial role in working memory and decision-making [191]. Recent studies have demonstrated how transformers outperform conventional models in detecting cognitive fatigue from EEG signals, providing a foundation for applications in performance monitoring and cognitive enhancement [192].

Seizure Prediction and Real-Time Applications.

AI models trained on continuous EEG streams have achieved remarkable accuracy in predicting epileptic seizures, a feat with profound clinical implications [193]. ML algorithms can identify preictal patterns up to 30 min before seizure onset, giving patients and caregivers critical time to prepare. Wearable devices powered by these AI models are enabling real-time seizure monitoring, transforming epilepsy management and improving patient safety [139]. The table below (Table 2) highlights significant studies showcasing AI’s impact on processing and analyzing neural signals.

### 5.3. Brain–Computer Interfaces: Toward Seamless Integration

BCIs are leveraging AI to create more adaptive and intuitive systems for restoring communication, mobility, and sensory feedback. These advancements are moving BCIs from research labs to real-world applications [202].

AI-Driven Neural Decoding.

AI has elevated the performance of BCIs by improving the accuracy of neural signal interpretation. DL models, such as CNNs, have enabled the precise control of robotic limbs, allowing users to perform complex movements like grasping objects or navigating environments. RL algorithms further enhance BCIs by adapting to individual neural patterns over time, reducing errors and improving user experience [8,203].

Integrating Sensory Feedback for Natural Interaction.

A major breakthrough in BCI development is the integration of sensory feedback systems powered by AI [204]. By interpreting neural activity, these systems provide users with real-time tactile sensations, allowing for more intuitive control of prosthetic devices. High-impact studies have shown that users equipped with AI-driven sensory feedback experience exhibited improved dexterity and task performance, marking a significant step toward seamless brain–machine integration [205]. Table 3 highlights key advancements in AI applications for BCIs, emphasizing their transformative potential in healthcare and beyond.

### 5.4. Computational Modeling: Simulating Brain Dynamics

AI-powered computational models are enabling the simulation of complex neural systems and offering insights into brain function, cognition, and disease progression. These models are instrumental in testing hypotheses and exploring new treatment avenues [215].

Simulating Cortical Oscillations.

SNNs, designed to replicate the brain’s time-dependent firing patterns, have advanced our understanding of neural oscillations. SNNs have been used to model inhibitory–excitatory balance disruptions, which play a critical role in conditions like autism spectrum disorders and epilepsy [101]. These simulations have revealed how altered synchronization in cortical circuits contributes to sensory processing deficits and cognitive impairments [216].

Predicting Neurological Disease Trajectories.

AI models integrating genetic, imaging, and clinical data are transforming our ability to predict the progression of neurological diseases. For instance, DL algorithms have simulated tau pathology spread in Alzheimer’s disease, identifying potential intervention points that could delay cognitive decline [217]. Similarly, predictive models of PD have linked dopamine depletion patterns to motor symptoms, guiding more personalized treatment strategies [218].

### 5.5. Drug Discovery and Development: Accelerating Therapeutic Innovation

AI is revolutionizing drug discovery by optimizing target identification, streamlining compound design, and improving predictions of therapeutic efficacy and safety. These advancements are particularly impactful in addressing the challenges of neurological drug development [219].

Identifying Novel Therapeutic Targets.

ML models trained on proteomic and transcriptomic data have identified key molecular pathways involved in synaptic dysfunction and neurodegeneration. AI has revealed regulators of amyloid beta aggregation in patients with AD and synaptic pruning in patients with schizophrenia, offering promising targets for early-stage interventions. These discoveries are enabling a shift toward precision medicine, where therapies are tailored to specific molecular profiles [220,221].

Designing Safer and More Effective Drugs.

GANs and RL algorithms are driving the development of novel compounds with optimized pharmacokinetic properties. A high-impact application demonstrated how AI-designed antiepileptic drugs exhibited reduced off-target effects while maintaining efficacy, accelerating their progression through preclinical trials [222]. These models are transforming the speed and efficiency of drug pipelines, particularly in conditions with complex pathophysiologies like epilepsy and multiple sclerosis [223].

### 5.6. Cognitive and Behavioral Analyses: Mapping Brain–Behavior Links

AI is advancing the study of cognition and behavior by uncovering connections between neural activity, adaptive processes, and mental states. These tools are shedding new light on decision-making, learning, and mental health [224].

Decoding Decision-Making Processes.

RL models have been used to explore how individuals adapt to changing environments, providing insights into reward-processing mechanisms. These studies have revealed how altered dopamine signaling affects decision-making in patients with conditions like addiction and depression. AI-driven analyses are informing the development of therapies aimed at restoring cognitive flexibility and improving executive function [225].

Personalizing Mental Health Care.

AI is transforming mental health diagnostics by integrating neuroimaging, behavioral, and physiological data. ML models have achieved high accuracy in predicting treatment responses, identifying patients likely to benefit from antidepressants or CBT [226]. These personalized approaches are reducing trial-and-error treatment strategies and improving patient outcomes. Furthermore, AI tools are being applied to detect early signs of conditions like PTSD, offering opportunities for timely interventions and preventive care [227].

## 6. Early Detection and Intervention Strategies Using AI: Case Studies

The integration of AI into early detection and intervention strategies has revolutionized neuroscience and clinical practice. By analyzing complex datasets, uncovering novel biomarkers, and enabling real-time decision-making, AI has opened new avenues for timely and precise healthcare [228]. This section explores key case studies, each illustrating how AI is transforming the diagnosis and management of neurological and psychiatric disorders, while maintaining a fluid narrative to highlight its unique contributions [229].

### 6.1. Early Detection of Alzheimer’s Disease: Multimodal Integration and Biomarker Discovery

AD presents significant diagnostic challenges, as its early symptoms often remain subtle and difficult to distinguish from normal aging. AI has addressed these challenges by integrating diverse data sources to uncover patterns that predict the onset of AD long before clinical symptoms appear [230].

In one groundbreaking application, AI models combined PET imaging, MRI scans, and cerebrospinal fluid (CSF) biomarkers to predict which individuals were at high risk of developing AD. By analyzing amyloid and tau deposition alongside structural connectivity disruptions, these models achieved predictive accuracies exceeding 90%, outperforming traditional diagnostic tools. Moreover, they provided novel insights into how early molecular changes interact with functional connectivity impairments in the brain’s DMN [231].

These advancements are reshaping the clinical landscape, enabling more precise patient stratification for clinical trials and facilitating earlier interventions. By focusing on those at the highest risk, AI-driven diagnostics are helping to delay or even prevent disease progression, making profound impacts on both individuals and healthcare systems [232].

### 6.2. Predictive Models for Seizure Management in Patients with Epilepsy

Epilepsy management has long been reactive, relying on a retrospective analysis of seizure patterns and limited by the unpredictability of episodes. AI has transformed this field by enabling real-time seizure prediction, providing patients with greater autonomy and safety [233].

One cutting-edge system utilized high-resolution EEG data and advanced neural networks to detect preictal patterns—subtle shifts in brainwave activity that precede seizures. By combining temporal and spatial features of brain activity, the model achieved seizure prediction accuracies of up to 90%, offering critical early warnings up to 30 min before onset. These capabilities were integrated into wearable devices, delivering real-time alerts to patients via smartphone applications [234].

This innovation has reduced seizure-related injuries and improved the quality of life of patients, enabling them to take proactive measures such as administering fast-acting medications or avoiding potentially dangerous activities. AI-powered solutions are fundamentally changing how epilepsy is monitored and managed, providing a new level of control to patients [235].

### 6.3. Autism Spectrum Disorder: Early Detection via Neuroimaging and Behavioral Analyses

Autism spectrum disorder (ASD) is often diagnosed late, missing critical developmental windows when interventions are most effective. AI has introduced novel methods for earlier detection by combining neuroimaging and behavioral data to identify early markers of ASD [236].

In a prominent study, ML algorithms were applied to fMRI data from infants with a high genetic risk of ASD. The model identified atypical connectivity patterns in brain regions associated with social communication, such as the temporal–parietal junction and prefrontal cortex. When these findings were combined with behavioral assessments, the AI system achieved diagnostic accuracy rates of 85% in children under two years old, far earlier than traditional diagnostic approaches [237].

By enabling an earlier diagnosis, these AI-driven tools allow clinicians to implement targeted interventions during critical periods of neural and social development. Personalized therapies focusing on language and social skills have been shown to significantly improve long-term outcomes for children with ASD [238].

### 6.4. AI-Driven Mental Health Diagnostics and Personalized Interventions

Mental health care is increasingly turning to AI for solutions to longstanding challenges, including delayed diagnosis and trial-and-error treatment strategies. By predicting treatment outcomes and personalizing interventions, AI is improving care for conditions such as depression and anxiety [239].

A notable study demonstrated how graph-based neural networks could analyze functional connectivity patterns in individuals with major depressive disorder [240]. By integrating these neural data with clinical histories and genetic markers, the model accurately predicted responses to selective serotonin reuptake inhibitors (SSRIs) with an 80% success rate. This capability has significantly reduced the reliance on guesswork, enabling clinicians to tailor treatments more effectively [241].

Beyond pharmacology, AI-powered platforms for CBT are revolutionizing mental health care. These systems adapt interventions based on user engagement and progress, delivering personalized support in real time. By increasing accessibility and tailoring care to individual needs, AI is breaking barriers in mental health care, particularly for underserved populations [242].

### 6.5. Stroke Care: Enhancing a Rapid Diagnosis and Treatment Precision

In stroke care, every minute saved in diagnosis and treatment can dramatically improve patient outcomes. AI has proven invaluable in this context, enabling faster, more accurate diagnoses and optimizing therapeutic decisions [243].

A pioneering AI model designed for stroke diagnostics analyzed CT and MRI scans in near real time, distinguishing between ischemic and hemorrhagic strokes with an accuracy of 95%. The system also quantified perfusion deficits and predicted tissue viability, ensuring that patients received appropriate treatments, such as thrombolysis, within the critical therapeutic window [244].

Hospitals integrating AI into their stroke care protocols have reduced door-to-treatment times by 25%, improving survival rates and minimizing long-term disability. These advancements underscore the transformative role of AI in emergency medicine, where speed and precision are paramount [245].

### 6.6. Parkinson’s Disease: Early Detection of Motor and Non-Motor Symptoms

PD is notoriously difficult to diagnose in its early stages, as its symptoms often overlap with those of other conditions. AI has addressed this gap by identifying subtle motor and non-motor signs that precede a clinical diagnosis [246].

A robust AI model trained on gait analysis, handwriting samples, and voice recordings achieved diagnostic accuracies exceeding 90%, detecting early motor impairments such as tremors and bradykinesia. By incorporating non-motor symptoms, such as sleep disturbances and a reduced sense of smell, the model provided a holistic diagnostic framework that traditional methods lack [247,248].

These advancements are enabling the earlier initiation of neuroprotective treatments, which can slow disease progression and improve quality of life. Additionally, AI models predicting disease trajectories are guiding clinicians in tailoring treatments to individual needs, making PD care more precise and effective [249].

### 6.7. Rare Neurological Disorders: AI for Diagnostic Support

Rare neurological disorders often go undiagnosed due to their complexity and the lack of widespread expertise. AI is bridging this gap by uncovering patterns in large datasets that point to rare conditions, offering diagnostic support where traditional methods fall short [250].

In a striking example, an AI system developed for diagnosing Wilson’s disease—a rare disorder of copper metabolism—analyzed genetic data, biochemical markers, and imaging findings to achieve a diagnostic accuracy of 92%. This tool significantly reduced diagnostic delays, which can span years, allowing for earlier treatment initiation and better patient outcomes [251].

AI frameworks like this are being applied to other rare neurological conditions, ensuring that even in resource-limited settings, patients benefit from early and accurate diagnoses. These advancements are democratizing access to specialized care, addressing a critical need in global health [252].

## 7. Challenges and Limitations

While AI is reshaping neuroscience and healthcare, its integration comes with a host of challenges that demand thoughtful solutions. From data quality and interpretability to ethical concerns and technical constraints, these obstacles must be addressed to fully realize AI’s transformative potential [2].

### 7.1. Data Quality and Accessibility

AI relies on high-quality data to function effectively, but in neuroscience, the quality and accessibility of data remain significant barriers [253]. Neuroimaging scans, electrophysiological recordings, and clinical datasets are often noisy, inconsistent, and influenced by varying acquisition protocols. These discrepancies can introduce bias and reduce the reliability of AI models, especially when applied across diverse populations [254].

Accessibility to large, representative datasets also presents a challenge. Many datasets are siloed within institutions or subject to strict privacy regulations, limiting their availability for collaborative research. In resource-constrained regions, where advanced neuroimaging and data collection infrastructure are scarce, this issue is even more pronounced [255].

Efforts like the Human Connectome Project and OpenNeuro are starting to bridge these gaps by providing open-access, standardized datasets, but much work remains [256]. Federated learning—a technique where AI models are trained across decentralized datasets without transferring sensitive data—offers a promising solution, enabling wider collaboration while respecting data privacy [257].

### 7.2. Interpretability of AI Models

AI’s strength lies in its ability to uncover complex patterns, but its “black-box” nature often leaves users questioning how it reaches its conclusions. This lack of transparency is particularly problematic in neuroscience and healthcare, where clinical decisions based on AI outputs can have life-altering consequences [258]. For instance, while an AI model may predict the onset of AD with high accuracy, its inability to explain the reasoning behind the prediction can erode trust among clinicians and patients [259].

To address this, XAI is emerging as a key focus. Techniques like attention mechanisms, saliency maps, and interpretable feature attributions are helping to make AI systems more transparent [260]. These approaches allow researchers and clinicians to better understand how AI models weigh different data inputs, ensuring that their outputs are not only accurate but also actionable and trustworthy [261].

### 7.3. Ethical and Privacy Concerns

The use of AI in neuroscience often involves handling sensitive data, such as neuroimaging scans, genetic profiles, and behavioral assessments. This raises pressing ethical questions about consent, data security, and potential misuse. Patients may be hesitant to share such intimate information, particularly if they fear breaches or misuse of their data [262].

Bias in AI models is another critical issue. If training datasets are not representative of diverse populations, AI systems can inadvertently perpetuate healthcare disparities, performing well for some groups while failing others. For example, models trained primarily on data from Western populations may struggle to generalize to patients from other regions or cultural backgrounds [263].

Addressing these concerns requires a robust ethical framework. Techniques like differential privacy, which protects individual data points within a dataset, and federated learning can enhance data security. Meanwhile, efforts to diversify datasets are essential to building AI systems that are equitable and effective across populations [264].

### 7.4. Technical and Computational Constraints

The sophisticated algorithms that power AI require significant computational resources, creating barriers for their adoption in resource-limited settings. Dl models, in particular, demand high-performance GPUs and extensive memory, which can be prohibitive for smaller clinics or research institutions. Additionally, training these models is both time-consuming and costly, limiting their scalability [265,266].

Neuroscience datasets, such as fMRI scans or EEG recordings, add another layer of complexity. These high-dimensional datasets require advanced preprocessing and optimization to avoid issues like overfitting, where models perform well on training data but fail to generalize to new cases [58].

Advances in cloud computing and edge computing are beginning to alleviate some of these challenges. Cloud platforms enable centralized processing, while edge computing brings AI capabilities directly to devices, reducing latency and resource demands. Lightweight AI models and transfer learning techniques are also making it easier to deploy effective systems in environments with limited computational power [267].

### 7.5. Integration into Clinical Practice

Despite AI’s potential, its integration into clinical workflows remains a significant hurdle. Many clinicians are hesitant to adopt AI tools, often due to concerns about reliability, interpretability, and disruption of established practices [268]. Additionally, regulatory frameworks for approving AI in healthcare are still evolving, creating uncertainty around compliance and accountability [269].

In practice, AI’s effectiveness often depends on its ability to complement, rather than replace, human expertise. For instance, while an AI system may identify subtle abnormalities in a neuroimaging scan, its insights must align with a clinician’s judgment to ensure an accurate diagnosis and treatment. Achieving this balance requires tools that are not only accurate but also user-friendly and seamlessly integrated into existing workflows [270].

Training programs for healthcare professionals are helping to bridge the gap, familiarizing clinicians with AI systems and their potential benefits. Regulatory agencies are also working to establish clear guidelines, ensuring that AI tools meet rigorous safety and efficacy standards before they reach clinical settings [271].

## 8. Future Directions and Opportunities

The future of AI in neuroscience holds extraordinary promise, with potential breakthroughs poised to revolutionize how we understand, diagnose, and treat brain disorders. By building on current advancements and addressing key challenges, AI will open uncharted frontiers in brain science [272].

### 8.1. Advances in AI Technologies

The next wave of AI innovation will feature advanced algorithms and architectures designed specifically for the complexities of neuroscience.

Explainable AI and Interactive Models.

A crucial future direction is the development of AI systems that not only generate accurate predictions but also interactively explain their reasoning [268]. For example, f (XAI) models could visually map neural activity patterns that lead to specific diagnoses, such as early Alzheimer’s, while quantifying their confidence in those predictions. These capabilities will enhance trust and adoption in clinical settings, bridging the gap between machine insights and human decision-making [273].

Revolutionizing Neuroscience with Generative AI.

Generative AI models, including GANs and transformers, are expected to play a pivotal role in neuroscience. These models could simulate realistic neural data, helping researchers test hypotheses and design experiments without relying on limited patient datasets [274]. A high-impact example includes GANs generating synthetic fMRI data to train AI systems for diagnosing rare neurological conditions, preserving patient privacy while improving model robustness [275].

Neuromorphic Computing for Real-Time Applications.

Neuromorphic systems, inspired by the brain’s parallel processing capabilities, will redefine AI’s efficiency and scalability in neuroscience. These energy-efficient models will enable the real-time processing of massive neural datasets, paving the way for applications like adaptive BCIs and continuous monitoring during neurosurgery [276].

AI for Synthetic Biology and Brain Organoids.

The intersection of AI and synthetic biology offers unprecedented opportunities to unravel the mysteries of the human brain. Brain organoids, lab-grown structures derived from stem cells, replicate many features of real brain tissue, serving as invaluable models for studying neural development, disease progression, and therapeutic responses. However, analyzing organoid data—spanning intricate gene expression patterns, structural imaging, and dynamic electrophysiological recordings—requires advanced computational tools [277]. AI-powered algorithms can help researchers efficiently process and interpret these complex datasets, uncovering hidden patterns that reveal key insights into neural development or the progression of neurodegenerative diseases. Generative models like GANs could simulate organoid growth and behavior, enabling researchers to run “virtual experiments” to predict how specific genetic mutations or drug treatments might affect organoid functionality [278]. These tools not only accelerate hypothesis testing but also reduce the need for time-intensive and costly laboratory procedures. With AI’s assistance, researchers could build personalized organoids to model patient-specific conditions, offering new pathways for understanding and treating neurological disorders [279].

Enhanced Human–Robot Interactions.

AI’s potential to improve the lives of patients extends far beyond diagnostics and therapeutic tools, particularly through its role in advanced human–robot interactions. Future humanoid robots, powered by AI-driven NLP and adaptive sensory systems, could act as highly personalized assistants for individuals with neurological impairments [280]. For researchers, these advancements could provide invaluable support in clinical settings, offering tools to monitor patient behavior, emotional states, and rehabilitation progress in real time. For instance, robots could analyze subtle behavioral cues, such as changes in facial expressions or body language, to detect stress or fatigue and provide real-time feedback to caregivers and clinicians [281].

In research environments, AI-integrated robots could assist in experimental procedures, adapting their interactions based on specific protocols or patient needs. For patients with severe motor impairments, such as those caused by ALS or spinal cord injuries, integrating AI with BCIs could enable the seamless and intuitive control of humanoid robots, fostering independence [282]. Beyond direct care, these systems could play a transformative role in cognitive rehabilitation and social engagement, providing researchers with tools to study long-term behavioral and neurological outcomes. By blending human–robot interactions with neuroscience, AI is poised to redefine the boundaries of assistive technology, offering solutions that are as functional as they are human-centered [283].

### 8.2. Personalized Neuroscience and Adaptive Therapies

The future of neuroscience lies in personalized approaches, where AI tailors diagnostics and treatments to the unique profiles of individual patients.

Dynamic Neurotherapies.

AI will enable closed-loop systems that deliver real-time, adaptive therapies for brain disorders. For example, deep brain stimulation (DBS) devices powered by AI will monitor neural activity continuously, dynamically adjusting stimulation parameters to optimize outcomes for Parkinson’s disease patients [284]. These adaptive systems will surpass today’s static treatments, reducing side effects while maximizing efficacy [285].

Precision Mental Health Interventions.

AI-driven tools will integrate neuroimaging, genetic, and behavioral data to provide hyper-personalized mental health care. For instance, AI systems could monitor changes in brain connectivity patterns during therapy sessions, adjusting interventions for conditions like depression or PTSD [286]. Highly cited research already demonstrates how ML can predict antidepressant responses, but future systems will take this further by recommending specific cognitive–behavioral techniques tailored to real-time patient feedback [287].

### 8.3. Multimodal and Temporal Data Integration

As neuroscience becomes increasingly data-driven, integrating information across multiple modalities and time points will unlock new insights.

Unifying Diverse Data Streams.

AI systems of the future will seamlessly integrate diverse datasets, from fMRI and EEG to genetic profiles and wearable sensor outputs. These multimodal models will provide a more holistic understanding of brain disorders, identifying connections that are invisible within single-modality studies [64]. For instance, combining EEG data with genetic risk scores for epilepsy could predict individual seizure patterns with unprecedented accuracy [288].

Tracking Brain Changes Over Time.

Temporal analysis will become a cornerstone of AI’s contribution to neuroscience. Longitudinal AI models will analyze how brain structure and function evolve across the lifespan, revealing the earliest signs of disorders like Alzheimer’s disease or schizophrenia. By detecting these subtle changes years before symptoms arise, AI will enable preventive interventions that preserve cognitive health [289].

### 8.4. Brain–Machine Interfaces: Next-Generation Applications

The integration of AI into brain–machine interfaces (BMIs) will expand their scope from assistive technologies to tools that enhance cognition and repair neural damage.

Neural Restoration Through AI-Driven Feedback.

Future BMIs will incorporate bidirectional communication, where AI not only decodes neural signals but also stimulates the brain to restore lost functions. For example, stroke patients could use AI-enhanced BMIs that provide motor feedback while training the brain’s plasticity, accelerating the recovery of movement and coordination [290,291].

AI-Enhanced Multisensory Prosthetics.

AI will drive the development of prosthetics that integrate vision, touch, and even auditory feedback, creating a seamless experience for users. ML algorithms will optimize these multisensory inputs in real time, allowing individuals to navigate environments and interact with objects as naturally as possible [292]. Highly cited studies on tactile sensing are already paving the way for these advancements, demonstrating how AI can simulate lifelike sensations in prosthetic hands [293].

### 8.5. Cognitive Augmentation and Neuroadaptive Learning

AI’s potential extends beyond treating disorders to enhancing human cognition and revolutionizing education.

AI-Driven Cognitive Enhancement.

AI will power tools that enhance cognitive functions like memory, attention, and decision-making. These systems will leverage neuroplasticity principles, offering personalized training programs based on real-time neural feedback. For instance, AI-guided interventions could help older adults maintain cognitive sharpness by targeting specific neural circuits linked to age-related decline [294,295].

Neuroadaptive Learning Platforms.

Education will be transformed by AI systems that adapt to individual learners’ neural and cognitive profiles. Neuroadaptive platforms could use wearable EEG devices to monitor engagement and dynamically adjust lesson difficulty, ensuring that students stay in an optimal learning zone [296]. These systems will be particularly impactful for individuals with learning disabilities, offering tailored support that maximizes their potential [297].

### 8.6. Ethical AI in Neuroscience

As AI becomes increasingly integrated into neuroscience, addressing its ethical implications will be critical to ensuring equitable and responsible progress.

Cognitive Privacy and Data Ownership.

As AI is capable of decoding neural activity, issues of cognitive privacy will become paramount. Future research must establish frameworks to protect individuals from the unauthorized use of their neural data, safeguarding the fundamental right to freedom of thought [298].

Equity and Inclusivity in AI Tools.

AI models often reflect the biases present in their training datasets, risking unequal performance across populations. Ensuring that future AI systems are inclusive and equitable will require concerted efforts to collect diverse, representative data [299]. Initiatives that prioritize fairness and inclusivity will help bridge healthcare disparities, ensuring that AI benefits everyone [300].

### 8.7. Collaboration and Global Impact

The future of AI in neuroscience will depend on interdisciplinary collaborations and global partnerships that accelerate innovation and expand access to advanced tools.

Interdisciplinary Research Teams.

The next phase of AI development will see the deeper integration of neuroscientists, computer scientists, clinicians, and ethicists working together to design robust and meaningful AI systems. Such collaborations will lead to tools that are scientifically rigorous, clinically impactful, and ethically sound [301].

Global Data-Sharing Initiatives.

AI’s potential can only be fully realized through global collaboration. Federated learning platforms will enable researchers from different regions to contribute data without compromising privacy, fostering discoveries that reflect the diversity of global populations. This approach will democratize access to cutting-edge AI tools, ensuring that their impact reaches underserved communities [302].

## 9. Conclusions

The partnership between AI and neuroscience is unlocking new horizons in understanding the brain and addressing its myriad complexities. This union is far more than a technological enhancement; it is a transformation that allows us to probe the mysteries of the human mind, improve diagnoses, develop tailored treatments, and explore untapped human potential. As AI continues to evolve, its role in neuroscience is becoming indispensable, opening pathways that were once unimaginable. This conclusion reflects on the journey covered in this review, highlighting key insights, the changing landscape, and the importance of collaboration for the future.

### 9.1. Summary of Key Insights

The integration of AI into neuroscience has yielded remarkable advancements, fundamentally reshaping the field. This review has detailed several pivotal contributions:

- Neuroimaging advancements—AI’s ability to analyze and integrate multimodal imaging data has revolutionized our understanding of brain structure and function. It has enabled the early detection of diseases like Alzheimer’s disease, mapped connectivity disruptions in patients with psychiatric conditions, and provided detailed biomarkers that traditional methods often missed.
- Enhanced neural signal processing—AI has significantly improved the analysis of neural signals from EEG, MEG, and other electrophysiological data. By leveraging sophisticated models such as transformers and neuromorphic computing, researchers can now decode real-time brain activity, predict seizures, and monitor dynamic neural states with unmatched precision.
- Personalized medicine—AI has propelled neuroscience into the era of personalized care. Tailored diagnostics and therapies, particularly in mental health and neurodegenerative diseases, have shown extraordinary promise in improving treatment outcomes and minimizing side effects.
- Breakthroughs in brain–computer interfaces—AI-powered BCIs have moved from experimental setups to real-world applications. These systems are restoring lost motor and sensory functions while offering exciting possibilities for enhancing human capabilities in ways previously thought impossible.
- Challenges addressed, opportunities unveiled—Despite its success, AI faces challenges such as data quality issues, interpretability, and ethical concerns. Innovations like XAI and federated learning are paving the way for more equitable, transparent, and reliable AI systems, ensuring broader and safer adoption.

### 9.2. The Evolving Landscape

The evolving relationship between AI and neuroscience is transforming the field from reactive to proactive, enabling earlier interventions and more precise treatments. Traditional neuroscience approaches, often limited by the complexity and volume of data, are being replaced by AI systems capable of revealing patterns and correlations that were previously hidden. These advancements are bridging the gap between basic research and clinical application, revolutionizing how we diagnose, treat, and understand brain disorders.

Beyond the clinical sphere, AI is expanding the scope of neuroscience into new territories. Neuroadaptive learning platforms, cognitive enhancement tools, and AI-driven neuroprosthetics are redefining what is possible in education, rehabilitation, and human augmentation. These developments signal a future where the brain is not only studied but actively optimized, creating opportunities to enhance cognitive function and improve quality of life.

AI’s democratization further amplifies its impact. Through global collaborations and equitable access initiatives, AI tools are being made available to underserved populations, ensuring that the benefits of neuroscience reach individuals worldwide. This democratization is fostering a more inclusive field where innovations address global health challenges rather than being confined to privileged regions.

### 9.3. Final Thoughts

The integration of AI and neuroscience is one of the most exciting frontiers in science, offering transformative potential that extends far beyond traditional boundaries. This partnership is not just about applying advanced algorithms to complex neural datasets; it is about reimagining how we understand and optimize the brain. Yet, the journey ahead requires more than technological breakthroughs; it demands ethical vigilance, inclusivity, and collaboration across disciplines.

Interdisciplinary efforts will be critical in addressing challenges such as cognitive privacy, data fairness, and equitable access to AI-driven tools. Scientists, clinicians, technologists, and ethicists must work together to ensure that AI’s capabilities are harnessed responsibly and for the benefit of all. Educating the next generation of professionals in the interplay between neuroscience and AI will also be vital, preparing them to lead in this rapidly advancing field.

Looking ahead, the possibilities are limitless. From unraveling the intricacies of brain function to transforming patient care and enhancing human cognition, AI’s potential to revolutionize neuroscience is boundless. This is not simply the conclusion of a review, it is the beginning of a new era, one where the mysteries of the brain are not only unraveled but leveraged to improve lives in profound and meaningful ways. By embracing innovation, fostering collaboration, and prioritizing equity, we can ensure that the future of AI and neuroscience is one of discovery, progress, and hope.

## Figures and Tables

**Table 1:** AI’s contributions to neuroimaging.

Reference	Study Objective	AI Methodology	Neuroimaging Modality	Condition Studied	Impact on Research or Practice
Liang et al. [181]	Predict fMRI responses to natural stimuli	Deep Learning (DNN)	fMRI	Visual perception	Enhanced decoding of visual cortical activity for brain–machine interfaces
Cui et al. [182]	AI-enhanced multimodal imaging fusion	GANs, Transformers	MRI, PET	Alzheimer’s disease	Improved the early diagnosis by integrating structural and molecular biomarkers
Luo et al. [183]	Multimodal imaging for ASD	Variational Autoencoders	MRI, fMRI	Autism spectrum disorder (ASD)	Linked functional connectivity disruptions to ASD-related behaviors
Khosla et al. [184]	Mood prediction from neuroimaging data	Graph Neural Networks (GNNs)	Intracranial EEG, fMRI	Mood disorders	Enabled predictions of mood variations from neural activity patterns
Saidi et al. [185]	Cross-modality Alzheimer’s disease detection	Convolutional Neural Networks	MRI, PET	Alzheimer’s disease	Demonstrated >90% accuracy in early-stage diagnosis using AI-based feature extraction
Gu et al. [186]	Functional connectivity mapping for schizophrenia	Dynamic Causal Models	fMRI	Schizophrenia	Identified disrupted brain network interactions associated with cognitive deficits
Nakack et al. [187]	Epileptic focus localization	Support Vector Machines (SVMs)	PET	Epilepsy	Improved the identification of seizure origins through molecular imaging data
Chen et al. [188]	Amyloid plaque detection with AI	GAN-enhanced PET reconstruction	PET	Alzheimer’s disease	Enhanced resolution and sensitivity for detecting early amyloid deposition
Ranjbarzadeh et al. [189]	Machine learning for brain tumor segmentation	U-Net Architecture	MRI	Brain tumors	Automated segmentation with >95% precision, facilitating surgical planning
Hu et al. [17]	Integrative imaging for neuroinflammation	Semi-Supervised Learning	MRI, PET	Multiple sclerosis	Identified inflammation-related imaging markers, aiding in treatment planning

**Table 2:** AI’s applications in neural signal processing.

Reference	Study Objective	AI Technique	Signal Type	Condition Studied	Key Results or Contributions
Kumar et al. [194]	Deep learning for BMI signal decoding	Convolutional Neural Networks	EEG	Motor imagery	Improved feature extraction for motor imagery, enhancing BMI control accuracy
Abduljaleel et al. [195]	Seizure detection using deep learning	CNN, RNN	EEG	Epilepsy	Achieved >90% seizure prediction accuracy, aiding in real-time management
Matar et al. [196]	Neural oscillation analysis for memory studies	Transformers	EEG, MEG	Memory and cognition	Identified oscillatory patterns linked to memory encoding and retrieval
Alessandrini et al. [197]	Neural correlates of Alzheimer’s disease via EEG	Semi-Supervised Learning	EEG	Alzheimer’s disease	Enhanced early detection through neural oscillation biomarkers
Azar et al. [198]	Real-time EEG analysis for emotion decoding	LSTMs	EEG	Anxiety, depression	Enabled real-time emotion monitoring for mental health applications
Norman et al. [199]	High-speed decoding of movement intentions	Reinforcement Learning	Electrocorticography	Paralysis	Improved accuracy in motor intention decoding for assistive BCIs
Jirsa et al. [200]	Cross-frequency coupling analysis of meditation states	Variational Autoencoders	EEG	Meditation	Identified theta–gamma dynamics associated with deep meditative states
Assali et al. [201]	Preictal state detection for epilepsy	GANs, CNN	EEG	Epilepsy	Improved sensitivity to detect seizure precursors, aiding preventative interventions
Tuncer et al. [192]	Cognitive fatigue detection from EEG	Support Vector Machines (SVMs)	EEG	Cognitive load	Developed real-time fatigue monitoring systems for high-stake tasks

**Table 3:** Advances in AI for brain–computer interfaces.

Reference	Study Objective	AI Technique	BCI Application	Condition Studied	Impact
Barnova et al. [206]	AI-driven BCIs for movement restoration	Reinforcement Learning	Movement control	Quadriplegia	Achieved seamless control of robotic arms for paralyzed users
Wei et al. [207]	High-accuracy SSVEP-based BCI	Canonical Correlation Analysis	Communication aids	General	Improved usability of visual BCIs for hands-free communication
Schiffer et al. [208]	Adaptive feedback for neurostimulation	Reinforcement Learning	Neurorehabilitation	Stroke	Accelerated recovery through real-time neuroadaptive feedback
Palumbo et al. [209]	Non-invasive BCIs for wheelchair navigation	Deep Learning Models	Mobility enhancement	Paralysis	Enabled thought-driven wheelchair navigation with high accuracy
Naik et al. [210]	Speech decoding via invasive BCIs	CNNs, GANs	Communication aids	ALS	Restored communication abilities for locked-in patients
Lin et al., 2023	Neuroplasticity-enhanced BCI adaptability	Neuromorphic Computing	Long-term adaptability	General	Improved long-term performance by incorporating neuroplasticity principles
George et al. [211]	Gaze-controlled BCIs for environmental navigation	Transformers	Accessibility tools	Paralysis	Enhanced gaze-driven BCIs, enabling navigation and environmental interaction
Maye et al. [212]	Multimodal BCI systems for sensory augmentation	Multimodal Learning Models	Sensory restoration	Amputees	Integrated tactile and auditory feedback for advanced prosthetics
Torres et al. [213]	Emotion-monitoring BCIs	SVMs	Mental health tracking	Depression, anxiety	Personalized mental health support through real-time neural monitoring
Moreno-Calderón et al. [214]	Gaming with thought-driven BCIs	GANs	Entertainment applications	General	Delivered immersive experiences controlled entirely by neural activity
